# Experiences of Users with an Online Self-Guided Mental Health Training Program Using Gamification

**DOI:** 10.1007/s41666-022-00124-z

**Published:** 2023-03-13

**Authors:** L. M. van der Lubbe, C. Gerritsen, M. C. A. Klein, R. F. Rodgers, K. V. Hindriks

**Affiliations:** 1 Computer Science, Vrije Universiteit Amsterdam, De Boelelaan 1111, Amsterdam, 1081HV The Netherlands; 2 APPEAR, Department of Applied Psychology, Northeastern University, Boston, USA; 3 Department of Psychiatric Emergency & Acute Care, Lapeyronie Hospital, CHRU Montpellier, Montpellier, France

**Keywords:** Mental health, Online training program, Gamification, User experience

## Abstract

Young adulthood is a period of high risk for the development of mental health concerns. Increasing well-being among young adults is important to prevent mental health concerns and their consequences. Self-compassion has been identified as a modifiable trait with the potential to protect against mental health concerns. An online self-guided mental health training program using gamification was developed and the user experience was evaluated in a 6-week experimental design. During this period, 294 participants were allocated to use the online training program via a website. User experience was assessed via self-report questionnaires, and interaction data for the training program were also collected. Results showed that those who completed the intervention (*n*= 47) visited the website on average 3.2 days a week, with a mean of 45.8 interactions during the 6 weeks. Participants report positive user experiences of the online training, on average a System Usability Scale Brooke (1) score of 79.1 (out of 100) at the end-point. Participants showed positive engagement with story elements of the training, based on an average score of 4.1 (out of 5) in the evaluation of the story at the end-point. This study found the online self-compassion intervention for youth to be acceptable, although some features seem preferred by users as compared to others. Gamification in the form of a guiding story and a reward structure seemed to be a promising element for successfully motivating participants and serving as a guiding metaphor for self-compassion.

## Introduction

Young adulthood is a critical developmental period. Experiences during these years have long-lasting implications on many aspects of adult life [2]. It is also a period of high risk for the development of mental health concerns. Almost half of the youth who will experience mental health concerns have started to develop symptoms by age 14, and among many such concerns may remain untreated into adulthood [3]. The burden of mental health disorders is high and associated with high mobility and impairment as well as negative impact on the immediate interpersonal environment [4]. For example, depression among college students can cause affective impairment or even academic impairment for moderate-to-severe levels of depression [3].

Given the course and consequences of mental health disordered among youth, their treatment and prevention are important areas of focus. However, many barriers to help-seeking exist among young adults. It has been suggested that young adults have a preference for self-help, find existing forms of help to lack accessibility and may experience difficulties with trusting the source of help [5]. Online interventions may help to overcome these barriers when they offer self-help because they are accessible as young adults are often experienced with using digital technologies, and there is no need to trust human training in fully automated interventions.

Self-compassion is an attitude of observant and non-judgmental self-kindness, and can simply be explained as compassion turned inward [6]. It has been shown that higher self-compassion related to positive well-being and mental health [7], and associated with psychopathology [8, 9].

Previously, an online self-guided self-compassion training was designed for young adults, and tested in a pilot study [10, 11]. In this training, gamification was added to engage participants in the training and to explain self-compassion and its uses in an attractive way, to increase their resilience in the face of difficulties such as stress and depression. This article describes a 6-week evaluation study of the online self-compassion training and presents findings related to user experience. The effects of the training on the mental health of participants are also measured during this 6-week study. However, these results will be published separately. This article explores how participants interacted with the system and the different components of the training.

In Section 2, background literature related to self-compassion and the mental health of young adults is presented. Section 3 presents the online training used for this study. Section 4 explains the research method applied in this study. In Section 5, the user experience of participants is described. Lastly, Section 6 draws conclusions about the user experience of the system and explores the research questions.

## Background

Self-compassion consists of three components: self-kindness, common humanity, and mindfulness [6]. Self-kindness (1) refers to being kind and understanding towards oneself instead of being self-critical. The notion of common humanity (2) refers to receiving your experiences as part of a larger human experience, and it is the opposite of being isolated. Mindfulness (3) involves a balanced view of negative emotions, opposite to over-identification with your negative emotions. Studies have shown that self-compassion is associated with many positive dimensions of psychological health and lower psychopathology [7–9].

Several in-person interventions have been shown to be successful in increasing self-compassion. The 8-week Mindful Self-Compassion (MSC) Program [12] is one of those interventions. The MSC program consists of 8 weekly 2-h group sessions, during which participants experientially engage with self-compassion through exercises and interpersonal group activities. The program has been shown to have positive effects on self-compassion, mindfulness, and well-being [12]. Digital interventions have also been studied, for example an app to support cancer patients by learning them about self-compassion [13] and the BodiMojo app for late adolescents and emerging adults to increase their self-compassion and body image [14]. Although these apps address the same topic (self-compassion), they do not use gamification elements.

Gamification can increase user motivation and user retention [15–17]. Gamification can be defined as ‘the intentional use of game elements for a gameful experience of non-game tasks and contexts’ [18]. A review study showed that in the mental health domain, most gamified applications targeted anxiety disorders and well-being [19]. Furthermore, this review showed that a justification for applying gamification could be found in 59% of the reviewed papers. The two main themes of justifications were as follows: promoting engagement with an intervention and enhancing an intervention’s intended effects. The most frequently used game mechanics were as follows: levels or progress feedback, points or scoring, rewards or prizes, narrative or theme, personalization, and customization. Although gamification is sometimes applied in interventions targeting mental health, the majority of the extant research comes from other e-health domains such as chronic disease management and rehabilitation and physical activity [20]. To the best of our knowledge, no interventions targeting self-compassion using gamification have been developed or evaluated. This article specifically focuses on points and narratives as forms of gamification and their application in an intervention targeting self-compassion.

To assess the global usability of a system in a reliable and low-cost way, the System Usability Scale (SUS) can be used [1]. This questionnaire includes 10 questions about the usability of a system that are rated on a Likert scale from 1 to 5. The total score of the SUS shows the overall system usability, which ranges between 1 (bad) and 100 (good). With this assessment, an overall evaluation of a system can be given, and it allows comparisons between different systems. Moreover, a bad user-experience was found to affect dropout in internet-delivered therapy [21]. Thus, it is important to know whether users have a acceptable user experience. Bangor et al. defined different ranges to classify SUS scores [22], such as the acceptability of scores and adjective ratings to describe a system. According to the scales presented by them, scores of > 70 are considered acceptable, scores 50–70 are considered marginally acceptable, and scores < 50 are not acceptable. Adjective ratings can be used to describe the SUS scores of participants. The following adjective ratings are described by Bangor et al.: < 38 is ‘worst imaginable’, 39–51 is ‘poor’, 52–72 is ‘OK’, 73–84 is ‘good’, 85–100 is ‘excellent’, and 100 is ‘best imaginable’.

## Self-compassion Training Website

An online self-guided self-compassion training intervention for young adults was previously developed and described in earlier work [10]. The training was accessible via a website, where it was always available. The intervention consisted of theory related to self-compassion, exercises to practise self-compassion, and supportive journals. These components are mainly based on the MSC Program [12]. While in the MSC Program the exercises and meditations are instructed by a professional, in this intervention the instructions were given by the system. Given this lack of professional oversight, the intervention was designed to try and minimize the potential for negative effects and mainly focused on positive elements (e.g. focus on self-compassion instead of stress or worries in the included journals), and none of the content was focused on negative emotions or experiences.

In a pilot study, the website was tested by participants from the target group (young adults) [11]. Based on the findings of the pilot, the intervention was changed and improved. See Table 1 for an overview of the content, exercises and journals in the definitive version of the intervention.

**Table 1:** Intervention content and new exercises/journals per week

Week	Theme and content	Exercises	Diary
1	**Introduction to self-compassion** - Components of self-compassion Additional material: - Mindfulness and self-compassion - Yin & Yang of self-compassion - Self-compassion is different from self-esteem - Which values are important to you?	**How to treat a friend** (based on an eponymous exercise from MSC Program): user reacts to a situation of a fictional friend, next they are asked how they would comfort themselves in the same situation. In the last step, they have to reflect on the differences.	**Choosing core values** (optional): users can choose up to 5 core values that they are reminded of in the header of the website
		**SC journal** (describing situations): describe daily life situations and how SC is applied/could have been applied in those situations (based on session ’how is MSC going for me?’ from MSC Program)	**Sky journal**: graphical journal to write about things that were positive, difficult or went from difficult to positive
2	**Self-compassion in daily life situations** - Misconceptions about self-compassion are used to further explain self-compassion Additional material: - Self-criticism and safety - Opening & closing	**Compassioned message** (based on ‘compassionate letter to myself’ from MSC Program): write a compassionate message to yourself, a good friend or to yourself as a good friend about a specific situation.	**SC journal** (complete version)
3	**Mindfulness**: - Focus on mindfulness component of self-compassion	**Mindfulness exercise**: semi-guided mindfulness practice	**Gratitude journal** (based on informal practice MSC Program about counting blessings): daily write about something you are grateful for.
4	**Practice with self-compassion** - Explains the stages of progress that participants can experience when increasing self-compassion - The structure of remaining training weeks	**Practicing self-compassion** (based on ‘motivate ourselves with compassion’ from MSC Program): write a compassionate message to motivate yourself in a difficult situation	

The theory of self-compassion was delivered in the form of a story (called tutorial on the website) that users progressed through by actively engaging in the training. A significant change from the initial design was the addition of weekly themes and additional content. Note that there are only themes unlocked for 4 weeks, while the study takes 6 weeks. This choice was made so that in the last 2 weeks, participants could practise without getting introduced to new exercises or journals. The metaphor that was used in the story remained the same after the pilot: self-compassion is explained with the metaphor of a journey.

The final version of the intervention included three exercises that remained unchanged since the pilot study (details on their design can be found in [10]), and a mindfulness exercise that was added after the pilot study. This exercise included instructions to practice mindfulness in the present moment or an example situation that can be encountered in daily life (e.g. ‘sitting outside in the sun’). Users practised this exercise on their own and made notes if they wanted.

Another aspect of the MSC Program that was included after the pilot was choosing core values. Users could choose up to 5 core values from a list, which were then shown on top of all pages to remind users what was important to them.

Additional changes were also made to the journals. First of all, the previously existing time restrictions to access the journals were removed. The remaining restrictions were that the gratitude journal could be completed once a day and that some exercises and journals were only available from a certain week and moment in the story. The restriction on the gratitude journal was set following the instructions in the MSC program to practise daily with gratitude. No other changes were made to the gratitude journal.

In the self-compassion journal, both the order and the content were modified to meet the requests of users from the pilot study. The new steps were that users first objectively describe a situation in their life, after which they indicate whether they used self-compassion or not and they were able to make notes about how self-compassion is connected to that situation. Next, instead of giving a rating, the mood journal was now integrated in this journal, and removed as a separate journal. Moreover, this step was made optional. Finally, users were able to save their journal entry as a favourite, which enables them to find the specific entry back easily. During the first week, only the first step (objective description) was available, to make users aware of situations that happen in their life that they might want to work on in the training without reflecting on it as they were just beginning to learn about self-compassion.

**Fig. 1:**
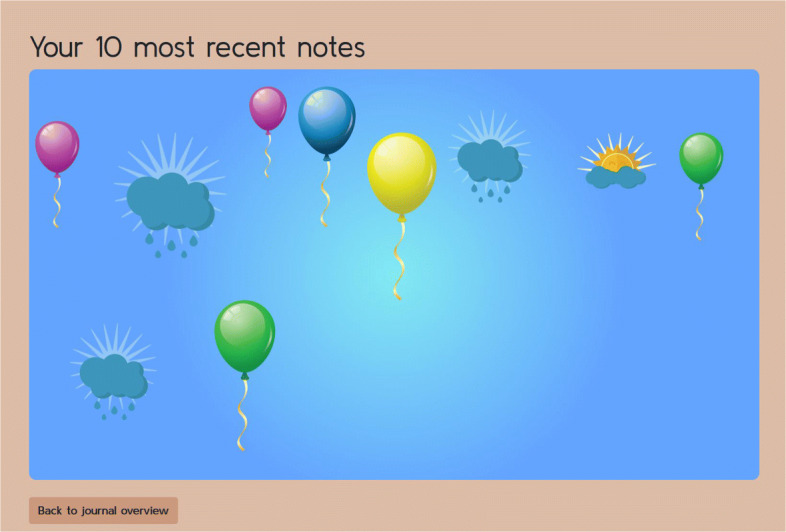
A screenshot of the Sky Journal overview page

A new journal was also added: the sky journal. In this journal, users could write about anything they had on their minds. The journal was visualized as a sky and users could put a balloon, rainy or sunny cloud in the sky (see Fig. 1, representing something positive that happened, something they were struggling with, and challenges that they faced but turned into something positive. Although users could write notes along with their visual choice, this was not mandatory.

The game mechanics used in the intervention did not change based on the pilot study. A new reward that was added was for completing the daily goal: a user received a bonus in the form of additional kilometres. In the first 3 weeks, the newly unlocked exercise of the week constituted that week’s daily goal. Moreover, once the gratitude journal was unlocked, this also became part of the daily goal. From week four on, the daily goal was a daily randomly selected exercise and the gratitude journal. Aside from creating more variety, the new daily goals reduced the time that users needed to interact every day, as there was a focus on only a few components instead of asking users to complete all components daily. Finally, the bonus appeared more graphically (with a pop-up with text and confetti) to make users notice it more prominently and increase their motivation to reach it.

Based on the findings of the pilot study, the focus of the training shifted from self-compassion as related to body image concerns towards improving well-being and life satisfaction in general by increasing self-compassion. As a result, the situations that were described in the narrative and exercises were broadened to reflect this more general focus. Based on the topics discussed by pilot participants, the following list of topics was created for exercise situations: social media, body image, social anxiety, relationships, job, school, and emotions. With the use of a specifically designed topic modelling algorithm [23], the topics discussed by users were detected in the gratitude and self-compassion journals and the ‘practise self-compassion’-exercise. For the two other exercises, the user was able to choose to either practice with a familiar, unfamiliar or random topic. Familiar topics were determined as the (maximum) 4 topics that were discussed by users according to the topic modelling algorithm.

Another small change that was made based on the pilot, was the colour theme of the website to make it look more appealing. There are multiple themes that users could choose from on their profile page.

## Method

First, this section introduces the research questions and how these were evaluated. After this, the design of the study, the participant numbers and characteristics, and the materials used in the study are explained in more detail.

### Research Questions

In general, this paper aims to give an overview of different aspects of the user experience. However, two aspects were of particular interest and therefore two research questions were developed regarding the user experience, based on the results of the previous pilot study with the system [11]. To explore these questions, only the participants who completed the end-point questionnaire were considered, since most data was available from them. However, their results were also compared to the results of participants that dropped out, to show differences between those who completed the intervention and those who did not. Since there was only limited data available from drop-outs, these results cannot be used to draw any conclusions.

The first research question was whether participants would be positive about the system’s usability. This research question was based on the experiences from the pilot study and the improvements that have been made after the pilot. The SUS scores of participants were used to explore this question. Moreover, answers from open-ended questions on the user experience were used to provide these scores with additional context. The evaluation of the system’s usability is considered positive if the SUS scores could be considered acceptable and could be described with an adjective rating of ‘OK’ or better (score > 52), according to the classification of Bangor et al. [22].

The second research question was whether participants would be engaged by the story element of the training. During the pilot study, participants indicated that they felt engaged and motivated by the story element of the training. Moreover, the story element is the most important gamification aspect of this training program. To explore this research question, questions about the story element were added to the mid- and end-point questionnaires.

The measures used for both research questions are explained in more detail in Section 4.4.

### Design

This article aimed to study user experience and engagement during the 6-week training. To study the user experience, participants were provided with access to the online training for the duration of 6 weeks. A repeated measure design was used: participants completed questionnaires about the user experience at mid-point and end-point.

As stated in the introduction, the mental health effects of the training were also examined in our study, but these outcomes will be described in another paper. The complete study is a between-groups design in which participants are randomly assigned to the training condition or control condition. The training condition meant that participants used the training. This condition is studied in this article. The control condition meant that participants could not access the online training, nor any other training provided in the study. For the mental health measures, the repeated measure design did not only include mid-point and end-point, but also a baseline measure.

### Participants

**Fig. 2:**
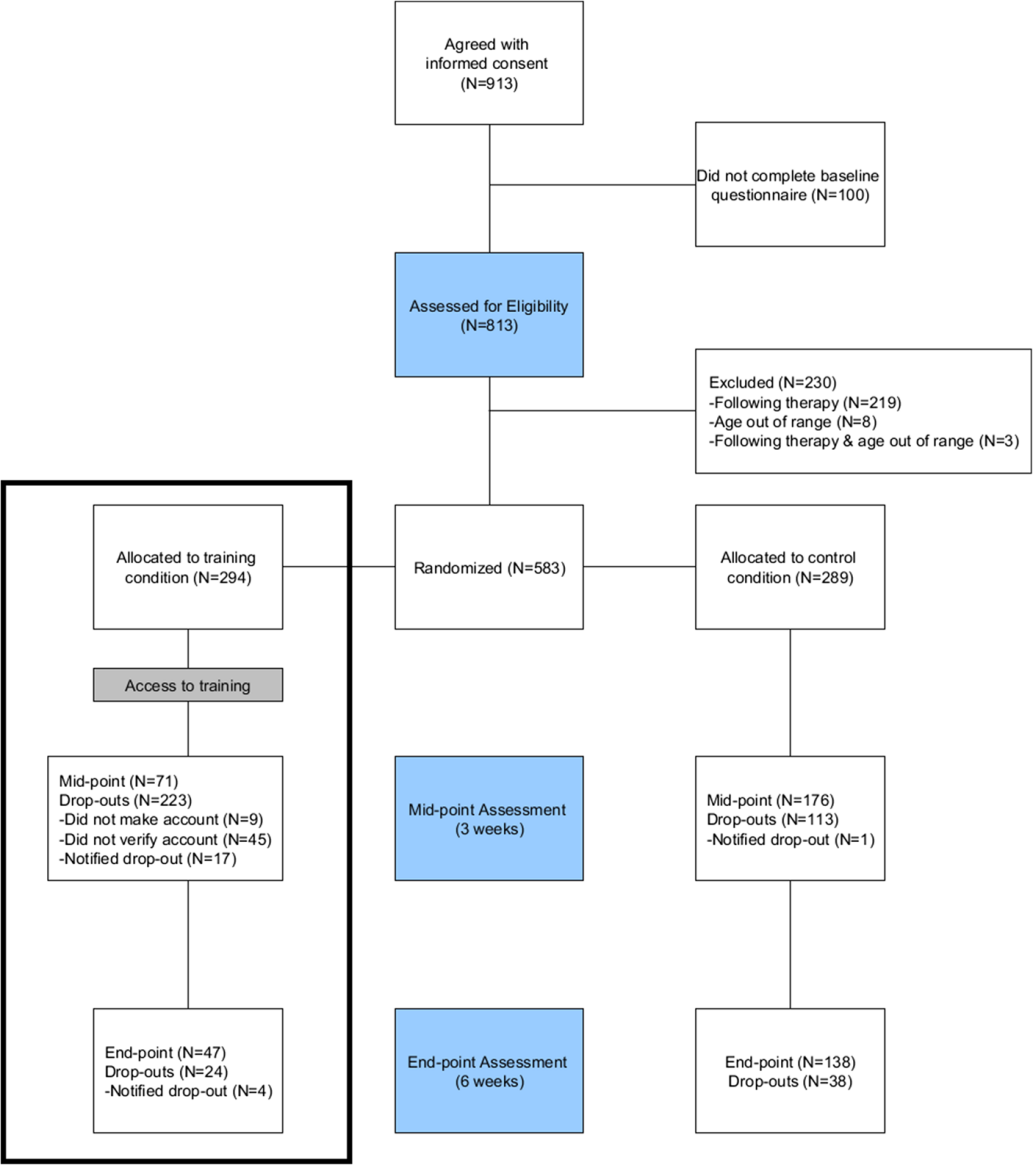
Participant flow—rectangle (left) shows participants discussed in this article

Figure 2 shows the participant numbers and the rates of drop-out in different phases of the experiment. Note that this also includes the control condition that is not reported on in this article, relevant participant numbers for this article are shown in the rectangle on the left. Inclusion criteria were that participants were not currently engaged in therapy for a mental health diagnosis (according to self-report) and were aged between 18 and 30 years old. Note that, although participants were assigned to one of the conditions randomly, the division of participants between the two conditions is not equal. This was caused by some participants signing up multiple times. In such cases they were removed from the control condition because it might be that they already used the training website.

Table 2 describes the demographics of the participants studied in this article (the training condition). The control condition will be describe in a future publication about the mental health effects, but this is out of scope for the current work.

**Table 2:** Baseline demographics for the training condition

	Training condition (*n*= 294)
Mean age, years (SD)	25.11 (3.381)
Gender, *n* (%)	
Female	279 (94.9)
Male	12 (4.1)
Other	1 (0.3)
Wish not to share	2 (0.7)
Highest education, *n* (%)	
Secundary education	11 (3.7)
Vocational education	19 (6.5)
Higher professional education	74 (25.2)
Scientific education (bachelor)	53 (18.0)
Scientific education (master)	137 (46.6)
Mean self-compassion score (SD)	3.22 (0.797)

In the training condition, 247 (84%) of the participants dropped out. The majority of this dropped out before the mid-point. This drop-out can partly be explained by the status of the user accounts of participants on the training website. Nine participants never created an account, and 45 participants did not verify their account, which together explains 21.9% of the drop-out. Moreover, 20 of the participants who dropped out completed the drop-out questionnaire (notified drop-out), which explains another 8.1% of the drop-out. One participant completed the drop-out questionnaire on the same day as (s)he received the final questionnaire, so this participant was not considered as a drop-out but did provide information about being inactive at the end of the training period. More details regarding the results of the drop-out questionnaire can be found in Section 5.3. Participants from the training condition (*n*= 47) that completed the intervention, completed all the questionnaires at the three time points.

### Materials

Participants in the training condition engaged in the training described in Section 3. Multiple measures were used to study different aspects of user experience and engagement with the online training. Mental health outcomes were also assessed, however, they are not reported on here.

#### Drop-out from Training

Once participants did not interact with the training for multiple days, they were asked whether they still wanted to continue. If they wished to cease their participation, they were asked to complete a short questionnaire about their reasons to quit. Various reasons for deciding to cease participation were assessed with open-ended questions and statements that were rated on a Likert scale. The questions constituting the drop-out questionnaire can be found in Appendix A.

#### User Experience

Several scales were included to assess the user experience of the online training. First of all, the SUS was used to evaluate the overall experienced system usability. Moreover, it is interesting to assess whether there are any significant relationships between the SUS scores and certain variables, such as the status of the participant (completing or dropped out), device used, age, self-compassion score at baseline and education level. This can provide us more information on whether specific groups need additional attention when designing future improvements to the training. Moreover, a set of questions was designed for this study specifically to assess aspects of particular interest related to the user experience (see Appendix B). Since these questions were specific for the context of this training website, no existing questionnaire was used. Based on the experiences during the pilot study, we created questions about the topics that were of our specific interest.

The first aspect was the device used for the training. This question was included to be able to explore the relationship between the device used and the SUS score, to see whether the website was less accessible via a certain device. Next, perceptions of the performance of the sentiment analysis used in one of the exercises was assessed. Furthermore, participants’ experience of the topic detection and topic choices for the exercises was evaluated. The story and journey elements were the most important gamification elements used in the training. Therefore, participants evaluated different aspects of these elements. This included perceived effects of the story on motivation, whether the story helped to understand self-compassion and the training, and how the story was experienced and delivered. Finally, participants were invited to respond to open-ended questions about lacking features or the user experience in general.

#### User Engagement

To analyze user engagement, log data from the website were used. These data contained information about exercises and journals completed by the users and information about the user profile such as which level in the tutorial participants reached and whether they chose their core values.

With the help of these data, analyses about the number of interactions and the type of user interactions with the system were performed. This gives information about which components of the website were used more often than others, and shows how engaged users are with the system.

### Procedure

The experiment was conducted completely online. Thus, informed consent and participant responses were all collected using the software Qualtrics. Recruitment took place via paid social media advertisement and through small personal recruitment. The social media advertisement was shown to users in the target age range in the Netherlands and the Dutch-speaking part of Belgium. In all cases, participants were first directed to the online information letter and informed consent. After providing consent, participants were given an identification number and they were redirected to the first questionnaire. This questionnaire started with demographics questions, to ensure that participants met the inclusion criteria. Included participants then continued with completing the other measures about their mental health. At the end of the questionnaire, participants were randomly assigned to either the training or the control condition.

Participants that were allocated to the training group could immediately create an account on the website and start with the training at any moment that is convenient for them. Three weeks after completing the baseline questionnaire (control condition) or after creating your account (training condition) a second questionnaire was sent (mid-point). This questionnaire included the mental health measures, but for the training condition, it also included the user experience measures mentioned above. After another 3 weeks, all active participants were invited to complete the same questionnaire again (end-point).

Reminders for the questionnaires were sent twice during the week after the initial invitation for the questionnaires. After that, participants were considered as drop-outs, unless they completed the questionnaire at their own initiative. When it was detected that a participant stranded in the study or training, for example the participant did complete the informed consent but did not complete the baseline questionnaire, reminders were sent as well.

Participating in the training could be seen as self-development, which was mentioned in the informed consent as one of the benefits of participating in the study. Afterwards, participants who were assigned to the control condition were granted access to the training as well, but they did not receive any more questionnaires and their data was not studied. Moreover, all participants received a debriefing information letter at the end containing information on how to continue to practise self-compassion. Participants who completed all questionnaires were entered into a lottery to win one of 10 online shopping vouchers of *€* 25 that were provided as a financial incentive.

## Results

User engagement and experience were examined using different statistics from the log data and the questionnaires. The research questions are explored in the second part of this section. Lastly, this section offers more insights into the drop-out of the training condition.

### User Engagement and Experience

#### Activity per Week

**Fig. 3:**
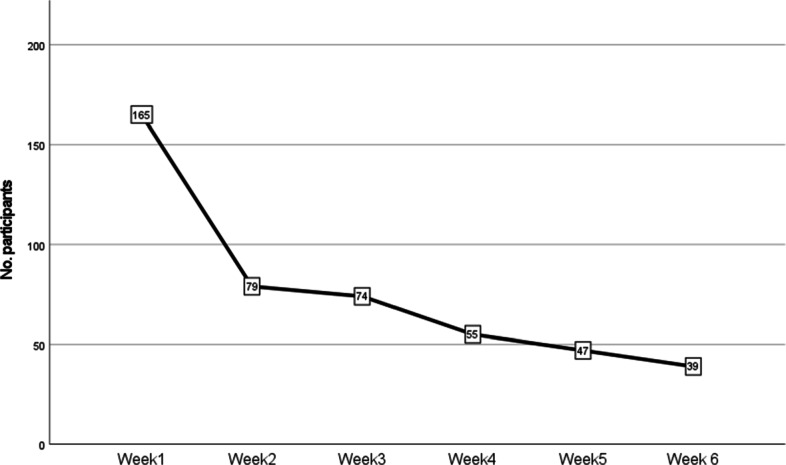
Number of participants active per week

Figure 3 shows how many participants were active at least once during every week of the training. Active means that at least one of the exercises and journals was completed. This figure shows that in the first week, only 165 of the 240 participants with a verified account (all steps of the sign-up process are completed) were active. However, in total 175 participants were active in any week, so 10 participants only became active after the first week. Moreover, 65 participants were not active in the training at all. The training was available until participants completed the final questionnaire. Some participants interacted with the system in the 7th week, before completing the final questionnaire. This concerned 20 participants with 1 or 2 days of interactions in the 7th week. These interactions are included in week 6 in the figures in this section.

Figure 3 only shows if participants were active at least once a week, and it should be noted this provides no information on how frequently participants interacted with the training. On average, participants (*n*= 240) were active during 2.26 days per week (SD = 1.365). Among those who completed the intervention (*n*= 47), the average was slightly higher: 3.17 days per week (SD = 1.521). Note that the 47 participants who were active in week 5 did not all complete the intervention. Completing the intervention was defined as completing the end-point questionnaire. It is a coincidence that the number for week 5 is also 47. Four outliers of participants that were active for more than 6 days a week were found. For those who did not complete the intervention and dropped out (*n*= 193), the average was as follows: 1.92 (SD = 1.136). For the average activity, the active weeks of the participant are used instead of the total training duration. If a participant had no activity at all, this participant was not considered in the averages.

**Fig. 4:**
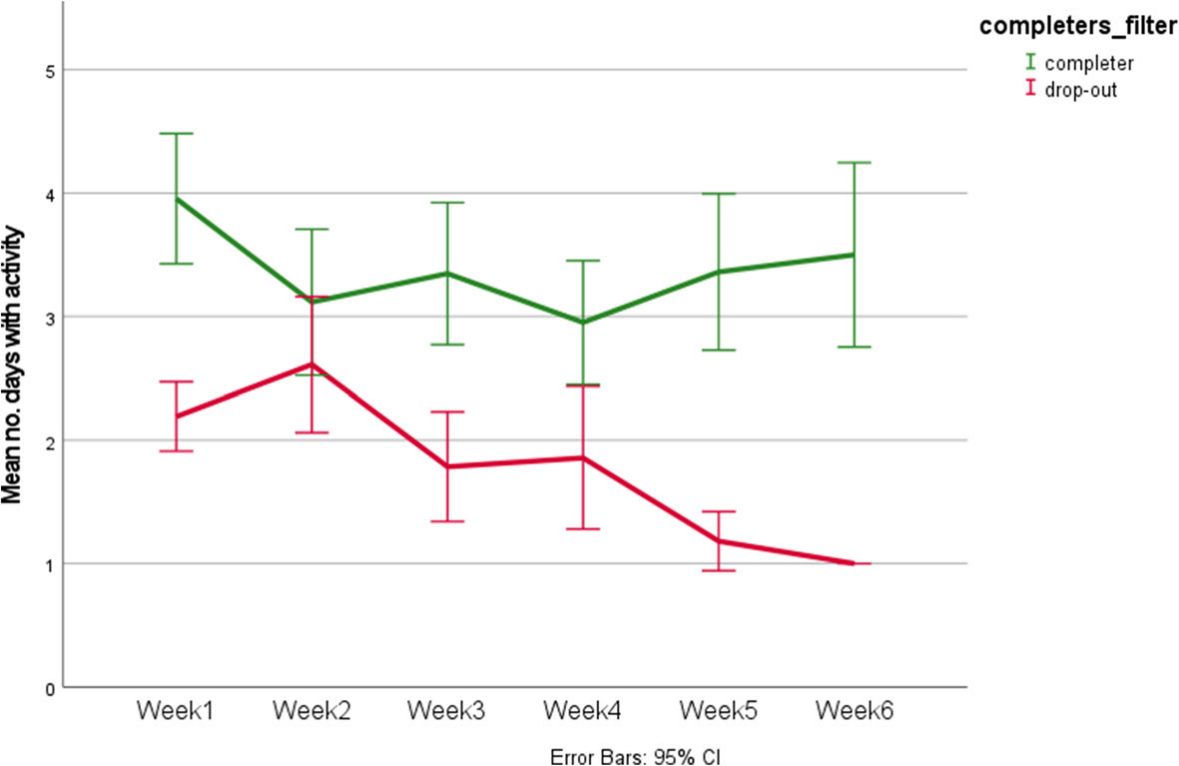
Mean activity per week

It was also of interest to see if participants become more or less active during specific weeks. Figure 4 shows the average activity for each week for those who completed the study and those who dropped out from the study. The figure shows that those who completed the intervention were more active compared to drop-out participants. This difference was statistically significant according to *t*-tests in all weeks, except for week 2. The activity of those who completed the intervention was the highest in the first week but remained quite stable over time. Among those who dropped out, activity decreased sharply after the second week.

#### Interactions Per Training Component

This section describes the number of interactions with the website per participant. An interaction referred to completing an exercise or journal. On average, participants (*n*= 240) had a total of 13.43 interactions (SD = 21.812) with the system over the duration that they were active on the website. However, a difference can be seen between those who completed the intervention and those who dropped out. The average total number of interactions of those who completed the intervention was 45.77 (SD = 27.300), while the average of those who dropped out was 5.56 (SD = 9.771). Although these numbers cannot be compared, since the period of activity is longer for those who completed the intervention and those who dropped out, it does show that those who dropped out of the intervention on average have a low number of interactions.

**Fig. 5:**
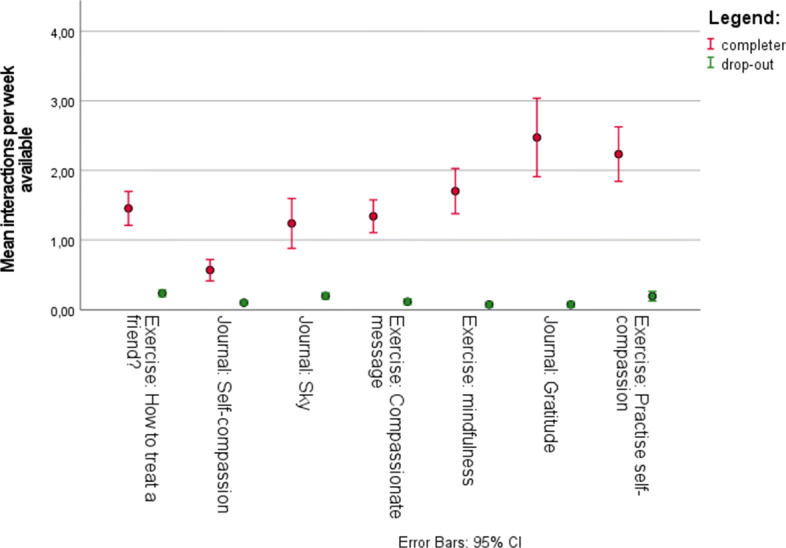
Interactions per program component, ordered on availability

To see which components were more popular among participants, the average number of interactions per week that a component was available can be studied (see Fig. 5). Note that in this graph no correction was applied when participants were not active in a week. This figure shows that among those who completed the intervention the gratitude journal was the most frequently completed, followed by the ‘Practise self-compassion’ exercise. The mean interactions per week available of the gratitude journal was statistically significantly higher compared to all other components, according to an ANOVA-test, except for the ‘Practise self-compassion’ exercise. The mean interactions per week available of the ‘Practise self-compassion’ exercise was statistically significantly higher compared to all other components. according to an ANOVA-test, except for the gratitude journal. The least frequently completed was the self-compassion journal, which was available the whole training. The mean interactions per week available for the self-compassion journal were statically significantly lower compared to all other components according to an ANOVA-test.

**Fig. 6:**
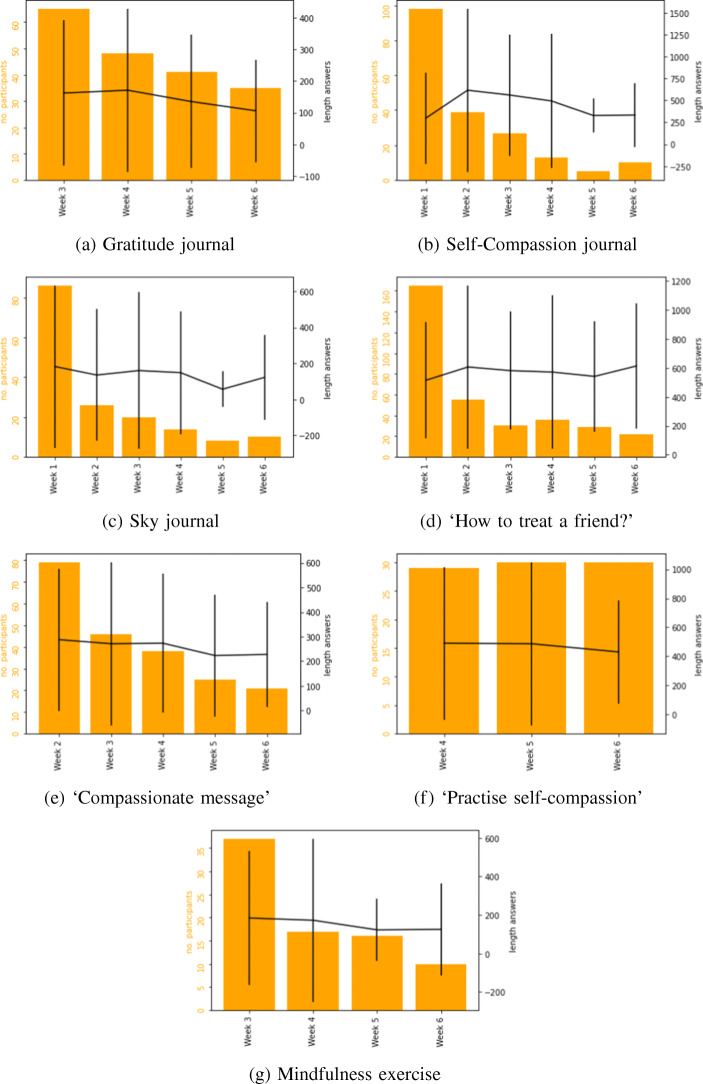
Answer lengths per training day

Figure 6 shows the mean length of answers given by participants (+ standard deviation), for all components of the training. In the orange bars, the numbers of participants with input for that component on a given day is shown. Note that occasional interaction in the 7th week is included in week 6 in the figures. Figure 6a shows that the number of participants slightly decreases over time. The length of answers in the gratitude journal remains stable over time with a slight decrease. Figure 6b shows the answers in the self-compassion journal. In the first week, only one part of the journal was available, after that week there was a slight increase in the length of answers after which it decreased a it again. Figure 6b also shows that the use of the journal quickly decreases. Figure 6c shows the notes in the sky journal. These notes were optional and were added for 50.5% of the journal entries. Empty notes are not taken into account for this figure, both for the answer length and the participant number. Although the lengths remain quite stable, the number of participants revealed a similar pattern to the self-compassion journal.

From Fig. 6d and e, it is clear that those exercises are most used in their first week, which is different from the exercise in Fig. 6f. This could be caused by the daily goal. In their first week, those exercises had been the daily goal, whereas the other exercise was not. For the mindfulness exercise, shown in Fig. 6g, the notes were optional. Only 41.3% of the completed exercises contained a note. Only completed notes are included in this figure for the answer length and participant numbers. The answer length of the notes remained stable, although for the number of users a similar pattern to the other exercises is observed. Overall, all components shown in these figures show quite stable answer lengths, and no clear trends emerged.

The additional material of the first week offered participants the possibility to choose core values, which they were reminded of during the whole training on the top part of every page. Of those who completed the intervention, 78.7% chose their core values, compared to 32.1% of those who dropped out.

#### Devices

Table 3 shows the devices participants reported to have used the most to access the training. Smartphones were the most popular devices among those who completed the intervention, but laptops/PCs were almost equally mentioned. Tablets were not used by those who completed the intervention. The drop-outs were using laptops/PCs slightly more often compared to those who completed the intervention. However, only a few of those who dropped out completed the mid-point questionnaire, so this view could be biased.

**Table 3:** Devices used by participants

	Drop-outs mid-point (*n*= 24)	Completers mid-point (*n*= 47)	Completers end-point (*N* = 47)
Laptop/PC	13 (54.2%)	21 (44.7%)	21 (44.7%)
Smartphone	10 (41.7%)	26 (55.3%)	26 (55.3%)
Tablet	1 (4.2%)	0 (0%)	0 (0%)

#### Sentiment Analysis

To assess how participants experienced the sentiment analysis in the ‘How to treat a friend?’-exercise, two questions were used. First, participants were asked whether they noticed that the last response in the first exercise was tailored to their previous answers in the exercise. At mid-point, most of those who completed the intervention noticed this (66%), while for those who dropped out only 41.7% noticed it. Participants mostly thought that the answers by the sentiment analysis matched somewhat with their own feelings: 50% of those who dropped out chose this answer and 63.8% of those who completed the intervention. At the end-point, 78.7% of those who completed the intervention noticed the sentiment analysis, and again most of the participants thought that it somewhat matched their feelings (68.1%).

#### Topic Detection

Two questions were included in the user experience questionnaire to assess the user experience with the topic detection functionality. Those who completed the intervention rated the question about whether the topics were suitable slightly above neutral (mid-point: Mean= 3.26, SD = 1.132, end-point: Mean= 3.23, SD = 1.108). The mean score of those who dropped out was not significantly different according to a *t*-test (*p* = 0.861): mid-point: Mean= 3.21, SD = 0.932. Those who completed the intervention (Mean= 4.26, SD = 0.706) are more positive about the question about how they liked the possibility to choose the type of situation compared to those who dropped out (Mean= 3.5, SD = 0.885). This difference was significant according to a *t*-test (*p*< 0.001). Those who completed the intervention rated this question the same at mid-point and at end-point.

#### User Feedback

Besides log data and closed questions, answers from two open-ended questions offered participants additional space to describe features they felt were missing or remarks they wanted to make. All answers from participants in the training condition at mid-point and end-point are read and summarized into different categories. In total, 44 answers (mid-point with completed intervention = 18, end-point = 18, mid-point dropped out of intervention = 8) of 27 participants (22 with completed intervention, 5 dropped out of intervention) were analysed.

Most remarks were about the fact that the website was sometimes unclear to users. They did not always know where to find information, how the tutorial worked or what was expected from them. Moreover, the user-friendliness of the website was sometimes considered insufficient, and some remarks about the clarity or structure of specific components were made. A suggestion made to improve this was to add a ‘frequently asked questions’ page to the website. It was noted during the coding that most answers by drop-out participants were about things that were unclear or not user friendly.

Another recurring topic was the tutorial. Participants did not like that it was split up into many texts, and could sometimes not read it properly when using their smartphone. It was noted that it was sometimes hard to find information in previous tutorials. A suggestion made to improve the readability was to add page numbers to the tutorial, which could make the progress clearer. Another suggestion made was to use an interactive video or cartoon instead of the tutorial, as this participant did not like all the reading. Despite these remarks, participants also answered that they were less motivated once the tutorial ended and that it was a pity that the tutorial ended halfway through the study.

Another topic that was discussed by participants was the automated responses of the system in exercises. They did not always fit and felt like standard responses. One participant noted that (s)he would have liked to receive a response from a professional.

Some participants described difficulties remaining motivated to engage with the intervention. Some participants explained that they missed the reminder emails because these were identified as spam in their email. One participant noted that once the routine of going to school was broken due to a COVID-19 lockdown (s)he forgot to log in. However, (s)he thought about the website in situations in which the training was applicable, so (s)he did return to the website for such events.

Different suggestions of additions to the website were made. Adding more guided mindfulness exercises, supported by audio or visuals/movies was proposed by participants. Other suggestions were to add more interactive exercises, more examples of compassionate messages, and transform the training more into a game. One participant emphasised that (s)he liked the training and would like to continue to use it.

### Exploring the Research Questions

#### Research Question 1: Participants Report Positive User Experiences of the Online Training

Among those who completed the intervention, the mean mid-point SUS-score was 75.69 (SD = 12.110). The mean end-point SUS-score was 79.10 (SD = 12.953). The mean SUS score for the mid-point of those who dropped out (*n*= 24) was 69.06 (SD = 16.316). The mid-point SUS scores of those who completed the intervention and those who dropped out were not significantly different (*p* = 0.087) according to a *t*-test.

The mean SUS scores found fall in the acceptable range of scores [22]. While the score of those who dropped out can be considered as ‘OK’, the ratings of those who completed the intervention can be classified as ‘good’. These results met the criteria mentioned in Section 4.1; thus, a positive attitude towards the system’s usability is found.

To test whether a relationship exists between the SUS score and certain variables, multiple regression analysis can be performed. Since 94.6% of the participants with an active account were female participants, separate analyses were not conducted among different gender. Multiple regression models were conducted to examine the variance in SUS scores at mid-point accounted for by completer status, age, education level, baseline self-compassion score and device used. Education level did not have a significant correlation with the SUS score. Therefore, education level was removed from the multiple regression model. The resulting model significantly predicted the mid-point SUS score, *F*(4, 66) = 2.631, *p* = 0.042. As presented in Table 4, higher age emerged as associated with lower SUS scores (*p* = 0.037). In addition, having completed the intervention versus having dropped out was associated with higher SUS scores (*p* = 0.094), and using a handheld (smartphone or tablet) was associated with higher SUS scores (*p* = 0.126). Self-compassion score at baseline (SC_baseline) does not seem the be associated with the SUS score (*p* = 0.781).

**Table 4:** Multiple regression results for SUS mid-point

SUS mid-point	*B*	95% CI for *B*		*SE B*	*β*	*R*^2^;	Δ*R*^2^;
		*LL*	*UL*				
Model						0.14	0.085*
Completers	5.71	− 1.01	12.43	3.36	0.196		
Device	4.95	− 1.42	11.32	3.19	0.179		
Age	− 1.16*	− 2.25	− 0.07	0.54	− 0.246*		
SC_baseline	− 0.676	− 5.51	4.16	2.42	− 0.32		

Model= ‘Enter’ method in SPSS Statistics; *B*, unstandardized regression coefficient; *CI*, confidence interval; *LL*, lower limit; *UL*, upper limit; *SE B*, standard error of the coefficient; *β*, standardized coefficient; *R*^2^, coefficient of determination; Δ*R*^2^, adjusted *R*^2^. *p< 0.05

#### Research Question 2: Participants have Positive Engagement with Story Elements of the Training

Table 5 summarizes the results of the questions on the story element of the training. The results showed that those who completed the intervention evaluated the story component *understanding* the highest, followed by the component *experience of the story*, both at mid-point as well as at end-point. The lowest score at the end-point was for the component on *delivery* and at the mid-point for the component on *motivation*. Since all aspects are on average evaluated with scores > 3, the engagement with the story element can be seen as positive.

**Table 5:** Evaluation story element: Mean scores per aspects (1-5)

	Completers mid-point	Drop-outs mid-point	Completers end-point
	(*n*= 47)	(*n*= 24)	(*N* = 47)
Motivation	3.92 (SD = 0.891)	3.06 (SD = 1.056)	3.95 (SD = 0.916)
Understand SC and training	4.35 (SD = 0.751)	3.64 (SD = 0.814)	4.38 (SD = 0.534)
Experience of story	4.19 (SD = 0.824)	3.56 (SD = 0.992)	4.21 (SD = 0.771)
Delivery of theory	4.06 (SD = 0.686)	3.30 (SD = 0.761)	3.88 (SD = 0.826)
*Total*	*4.12 (SD = 0.610)*	*3.38 (SD = 0.772)*	*4.08 (SD = 0.617)*

Those who completed the intervention were more positive compared to those who dropped out, who endorsed all of the aspects significantly lower as compared to those who completed the intervention. This difference was significant for the subscales as well as the total score and is tested using *t*-tests.

### Drop-out

The answers to the questions from the drop-out questionnaire (*n*= 21) were analysed and coded into five categories: technology of the training, content of the training, time investment, (mental) situation, and other. Eight participants indicated that they did not have time or that the training took more time than expected. Three participants mentioned that they chose to crease participating due to mental health concerns. Two participants mentioned other reasons such as not needing the training and forgetting about it. Eight participants in total mentioned something related to the training itself. For 3 participants the technology used was a reason for leaving the study, either because they did not like the way it worked or because they experienced problems, the other 5 participants commented about their appreciation for the content of the training, for example that they were not motivated by it, did not like the exercises or had other means of doing the same things (for example gratitude journal).

Below are the average scores for the predefined reasons, scored 1–5 (no reason for quitting - important reason for quitting) (*n*= 20):

- The study/website was not interesting: Mean= 1.75, SD = 1.372
- I didn’t like participating: Mean= 2.10, SD = 1.294
- Participating costs too much time: Mean= 3.15, SD = 1.585
- The website was not useful for me: Mean= 2.15, SD = 1.387
- I thought it was emotionally heavy to participate: Mean= 1.25, SD = 0.639
- The website did not work properly: Mean= 2.05, SD = 1.605
- I didn’t understand the website: Mean= 2.05, SD = 1.276

The results from these questions suggest that participants often felt they lacked the time to complete the intervention, or that the intervention was too time-intensive. However, none of the other reasons seemed to be a prominent reason for quitting. These findings suggest that a combination of factors might be responsive for individuals ceasing to participate, or that the most important reason was not included in the questionnaire. Another important reason was technical problems with the website or a lack of enjoyment.

In addition, participants were asked (*n*= 21) what could have been done differently. Ten participants said that nothing could have been changed, that it was their problem, or did not indicate anything. Two participants thought that the time investment could have been clearer from the start. The remaining 9 participants made comments about improvements to the (accessibility of the) website and the training, such as increasing the performance on mobile devices and allowing users to listen to texts instead of reading them.

The self-compassion scores of participants who completed the study were compared with those that dropped-out to see whether the initial self-compassion score could have influenced their compliance. This difference was found to not be statistically significant *p* = 0.092.

#### Training Progress and Drop-Out

The narrative with the theory about self-compassion (called the tutorial) was divided into steps that users have to click through. It can be studied in more detail when participants dropped out, by looking at the step in which they stranded. Table 6 shows the number of participants and their final tutorial level. The 129 participants that dropped out in the first week include the 65 participants that never had an interaction with the system. This shows that most of the drop-outs happen in the first week of the 4-week tutorial, but there is also some drop-out among participants that completed the tutorial. Among those who completed the intervention, it can be observed that 5 participants did not continue with the tutorial after the 3rd week of the training; however, they still completed all questionnaires.

**Table 6:** Participants and their tutorial levels

	Drop-outs (*n*= 193)	Completers (*n*= 47)
Week 1 (level < 32)	129 (66.8%)	0 (0%)
Week 2 (level > 31 and level < 55)	31 (16.1%)	0 (0%)
Week 3 (level > 54 and level < 66)	16 (8.3%)	5 (10.6%)
Week 4 (level > 65 and level < 75)	0 (0%)	0 (0%)
Tutorial complete (level > 74)	17 (8.8%)	42 (89.4%)

## Discussion and Conclusion

This study explored the engagement of users with an online self-compassion training website. Overall, our findings indicated that, similar to other online self-compassion based programs [14, 24, 25] users engaged with the program and reported positive experiences. These findings are promising in terms of the uptake of such programs among university students. For those who completed the intervention (*n*= 47), the most frequently completed activity was the gratitude journal, even though this was only unlocked in the third week. This was followed by the exercise called ‘Practise self-compassion’. The self-compassion journal was completed the least often, although it was open to users since the first week. Figure 6b shows that after the first week, the interaction with the journal reduces. Even when in the second week more steps were added to the journal, this did not seem to increase the interaction with the journal. On average, those who completed the intervention had 45.77 (SD = 27.300) interactions on the training website during the full study. Other authors have suggested that completing exercises that increase skills may be more important than interacting with the online program itself [25]. Insights into which activities were particularly engaging to the users is important. It may be that strategically introducing activities that are known to be engaging over the course of an intervention could be a good means of increasing retention.

The first research question of this study was whether participants would report positive user experiences of the online training. Based on the SUS scores reported by participants, this was supported. Although remarks about possible improvements in the functioning and clarity of the website were made by participants, the overall usability of the system is acceptable. The high acceptability of the intervention is an auspicious finding. It is likely that the previous work conducted to refine the pilot intervention with the help of user feedback increased this satisfaction [11]. User satisfaction is a critical element for maintaining engagement and therefore these findings suggest that the current intervention would be an useful tool for promoting mental health among young adults [26].

The second research question was whether participants would engage positively with story elements of the training. From the results, it seems that the story was mainly valued for explaining what self-compassion is and what can be expected from the exercises and journals. Based on the pilot study, it was expected that participants would mainly appreciate the motivation aspect, but this turned out to be not the most highly valued aspect [11]. The story contributed to the understanding of participants, and it thus was an important aspect of the training. Moreover, since all aspects of the story were evaluated very positively by those who completed the intervention, this study has shown that a story is a suitable and valuable game mechanic to add to a mental health training intervention, which is consistent with previous work [27].

A large drop-out was noted in this study: only 47 participants (16.0%) of the training condition completed the study. However, half of the drop-out occurred before any interaction with the website took place. In the absence of any interaction with the website, these rates of drop-out cannot be interpreted as reflecting on the quality or the content of the training. It could be that the procedure was not clear to them, or they did experience startup problems. There is however no data to support this conclusion. It should also be noted that drop-out is a recurrent issue in online mental health interventions, and further innovative solutions to prevent this are needed throughout the field [28].

Apart from these non-starters, the data on the tutorial levels of participants showed that 64 participants did not move beyond the content of the first week. Together, the participants without a (verified) account and the participants that left the intervention during the first week make 74.1% of the drop-out. After finishing the first week, 42.3% completed the whole study. These findings regarding the timing of drop-out suggest that perhaps additional clarification of study goals and expectations might be helpful to ensure that participants fully complete the intervention. Alternatively, it might be useful to understand what expectations were not met so as to evaluate the needs of young adults who might identify such an intervention as useful but not benefit from this specific format.

The recruitment, sign-up, and instructions for the training were all automated. Moreover, a paid advertisement was used, reaching a broad audience. This could have attracted a less intrinsically motivated group of participants, which could have caused the high drop-out before interacting with the website. Once participants were engaged in the training, the drop-out was much lower. Often drop-out happened between 2 weeks in the tutorial. It could be that participants forgot to return after that, or that it is unclear to participants what to do and expect later. There were data to support this: from analysing the open-ended questions, it became clear that comments made by participants who did not complete the intervention mostly pointed to elements that were unclear or not user friendly. This could be related to the high drop-out at the beginning of the training. Moreover, problems with automated reminders ending up in spam filters could have also contributed to the drop-out. It has been highlighted that appropriate incentives and intervention brevity may be key in keeping youth engaged in such interventions [29].

Regarding participants that remained active in the first weeks but dropped out later, it can be observed that they were generally less active and less positive about the training as a whole. This can be seen in Fig. 4, where the mean weekly activity of participants who dropped out was lower compared to that of the participants who completed the study. Participants who dropped out have, on average, 5.56 (SD = 9.771) interactions in the training. Participants who dropped out evaluated the training website less positively, evaluated the story less positively on all aspects assessed and were less positive about the sentiment analysis and topic detection element. Thus, satisfaction with the intervention as a whole was associated with the likelihood of completing the intervention or not, which is consistent with previous findings and highlights the importance of user experience [29].

Although the recruitment did not target a specific gender, the resulting sample of the trining group consists of 94.4% female participants. It is therefore unclear how the found results generalize to a male population. However, this could also indicate tht mostly female users are attracted to such an online mental health program. Future research could look into this in more detail.

A limitation of the intervention was that most content and activities were textual. Using different media, such as audio or video, could have attracted more users and could have made the training more accessible to a wider range of users. Broader dissemination of the intervention to a wider audience would be important in future work. Thus, incorporating different types of media would be an improvement to consider.

The number of game elements used was also limited. The focus was on the story and the rewards contributing to the story. However, other game mechanics could also be added, or the current mechanics could be extended, as well as including more variety in terms of media types. For example, if more visuals were used, it would be possible to add more customization options (such as character creation) and add visual collectables (such as pets or accessories for your avatar). Although desirable, these elements were beyond the scope of the current work. However, moving forward it would be useful to incorporate them in interventions to further improve the user experience.

Another limitation was that the research questions were explored using only the data from those who completed the intervention. Since the majority of drop-out occurred before the study mid-point, only limited data are available from the participants who did not complete the intervention. If more data had been available, it would have been possible to explore the research questions by using both the data from those who completed the intervention and those who dropped out.

The second research question focused on the story element, which was the most prominent game mechanic used. However, the other elements (rewards and customization) were not assessed separately. However, as they were closely related to the story element, it can be presumed that participants might also have been positive about those elements. A limitation of this study is therefore that it was not possible to determine the effect of the individual elements. However, this is often difficult, as game elements are hard to isolate and their meaning and effect can change if isolated.

The large numbers of participants who left the study between the initial registration and the end of the first week revealed that the onboarding for such a program was a challenging, yet important aspect to consider. Future work should consider tools like notifications or text message reminders to increase participant retention [28]. Another possibility would be to integrate the training website into a program structure in which a professional could be involved. If participants feel that they are part of a larger program and that there is some supervision, this might increase the adherence. However, this could create a barrier to joining such a program, and is substantially more onerous in terms of resources. It would be interesting to study the effects of integrating the training website into a supervised program, for example in collaborations with schools, colleges, or universities.

Findings related to the topic detection algorithm suggest that participants like and see potential in personalization by choosing if they want to practise with topics they have previously discussed or not. However, the feedback from participants also suggested that this aspect would benefit from further improvement. It should be noted that it could also be that participants did not notice the topics, or did not pay attention to the accuracy. Since participants did like the potential of the algorithm, future research should look into (improving) the accuracy of the algorithm.

Another promising direction for future research would be to extend and improve the narrative in the training. As participants were enthusiastic about this aspect of the training, it would be beneficial to explore this further. As mentioned, adding different media into the story could improve the user experience. Other options would be to extend the content of the story or to add more interactivity to the story, for example allowing participants to choose which topic to learn about in the next story part.

To summarize, this study found the refined online self-compassion focused intervention for youth to be acceptable, although some features seem preferred by users as compared to others. Despite the positive ratings of the intervention, retention was not high. However, the majority of participants were lost before the intervention was initiated or during the very first week suggesting that this was not related to aspects of the intervention itself. Gamification in the form of a guiding story and a reward structure seemed to be a promising element for successfully motivating participants and serving as a guiding metaphor for self-compassion [30]. Future work is needed to best disseminate such interventions to target populations and to further improve designs to best retain and engage participants.

This article contributes to understanding how self-guided mental health applications can be build and how they are used by participants. Moreover, it shows how gamification can be added to a training program about self-compassion. Future research in similar directions can benefit from the examples and experiences described in this article.

## Appendix : A: Drop-out questionnaire

- I want to quit the self-compassion study. This means that I can no longer use the training website and I will no longer receive questionnaires. [Yes I quit the study, No continue with the study]
- Why do you want to quit the study? [open]
- Below you find a list of reasons to quit the study. Please indicate to what extent these reasons played a role in quitting the study. [Likert 1-5 (played no role - played an important role)]
  - The study/website was not interesting.
  - I didn’t like participating.
  - Participating costs too much time.
  - The website was not useful for me.
  - I thought it was emotionally heavy to participate.
  - The website did not work properly.
  - I didn’t understand the website.
- What could we have done differently so that you would not have dropped out? [open]
- Any other remarks? [open]

## Appendix : B: User Experience Custom Questions

All questions are translated, the original questionnaire was in Dutch.

### B.1: Device Used

- Which device did you use most often to visit the website? [Laptop/PC, Smartphone, Tablet]

### B.2: Sentiment Analysis

- In the exercise ‘How would you treat a friend?’ we used techniques to adapt the answers from the system to your answers. Did you notice this during practice? [Yes, No, I don’t know]
- Take a look at your answers in the ‘How would you treat a friend?’-exercise. Looking at the last answer of the system, do you feel that it matches your answer? [Likert scale 1-5 (do no match at all- match very well)]

### B.3: Topic Detection

Topics were automatically added to some exercises and journals. Based on those topics, you could choose to practise with familiar or unfamiliar situations in different exercises. To which degree do you agree with the following statements? [Likert 1-5 (totally disagree - totally agree)]

- The automatically detected topics match with the topics I discussed in the journal/exercise.
- I liked that I was able to choose which type of situation I wanted to practise with.

### B.4: Story

To explain what self-compassion is about, we used a story (tutorial) in the first 4 weeks of the training. After those 4 weeks, you could still read parts of the story when you reached a destination. Indicate to what extent you agree with the following statements: [Likert 1-5 (totally disagree - totally agree)]

- The story motivated me to remain active throughout the training.
- The story helped me to understand self-compassion.
- I liked to read the story.
- Each week’s story had a nice length (not too long or short).
- I liked that the story was divided over 4 weeks.
- I understood the exercises and journals better because of the story.
- The story matched with my world of experience.
- I wanted to progress in the journey and this motivated me to do exercises and fill in journals regularly.
- I reached new destinations often enough.

### B.5: Other Questions

- Did you miss any functionalities on the website? [No, Yes namely (open)]
- Do you have any other remarks? [open]
